# Comprehensive perioperative and minimally invasive post-operative defect approach of massive palatal pleomorphic adenoma: Case series

**DOI:** 10.1016/j.ijscr.2024.110208

**Published:** 2024-08-25

**Authors:** Nining Dwi Suti Ismawati, Andreas Pratama Nugraha, Ronny Baehaqi, Irham Taufiqurrahman

**Affiliations:** a Oral and Maxillofacial Surgery Division, Dr. Soetomo General and Academic Hospital, Surabaya, Indonesia; b Department of Oral and Maxillofacial Surgery, Universitas Lambung Mangkurat, Banjarmasin, Indonesia; c Medical Faculty, Universitas Airlangga, Indonesia

**Keywords:** Pleomorphic adenoma, Maxillary, Minimal invasive, Palatal defect

## Abstract

**Introduction and importance:**

Extensive Palatal post-operative defect management following excision of neoplasm is one of the most difficult challenges for oral and maxillofacial surgeons regarding its limited surgical access and visibility on narrow area, airway management difficulty during intubation, richness of maxillary vascular network resulting in enormous bleeding risk. Decision making regarding its surgical approach and impact on speech and mastication is important. This case series aim to describe comprehensive step by step perioperative and palatal defect management approach based on tumor pathological characteristic and anatomical perspective to achieve good surgical outcome.

**Cases presentation:**

Two cases of massive palatal pleomorphic adenoma were presented. Both of cases occurs in female patients. Lesions was crossing the midline, impair speech and causing discomfort. Preoperative diagnostic from CT scan and FNAB result was pleomorphic adenoma.

**Clinical discussion:**

Surgery for both cases done with wide periosteal sacrificing excision, ostectomy and surgical obturator placement from intraoral approach under general anesthesia with nasal intubation. Eventually the wounds healed without wound dehiscence and fistula, no speech impairment and no sign of reccurency.

**Conclusion:**

Understanding pathological characteristic of pleomorphic adenoma and basic anatomy of surrounding structure are important to formulate minimal invasive surgical and post-operative defect management planning and improve patient's quality of life.

## Highlight

• Pleomorphic adenoma in palate with enormous size was treated with minimal invasive surgical approach
• Treatment approach was based on deep comprehension of basic medical science such as wound healing mechanism
• Local ostectomy was done instead of infrastructure maxillectomy to preserve vital structure
• Obturator was placed to protect the underlying secondary wound healing and achieve excellent wound healing

## 1 Introduction

Pleomorphic adenoma is a rare benign mixed tumor classified by the World Health Organization as Salivary gland tumors which comprise 3–5 % of all head and neck tumors. It was one of the most common salivary gland tumor representing approximately 45–75 % of salivary grand tumor cases, with an annual incidence of 2.4–3.5 cases per 100,000 inhabitants [1]. Pleomorphic Adenoma mostly occurred at parotid glands (85 %) followed by minor salivary glands in hard palate, soft palate and upper lip (10 %) and the submandibular glands (5 %). Adult females in the third to fifth decade are most commonly affected [2].

Intraoral pleomorphic adenoma is a rare entity, with the most common site of appearance being the palate because highest count of 450–750 minor salivary glands are present on palate, particularly at the junction of hard and soft palate [[3], [4], [5]]. Regardless of the site of origin, the pleomorphic adenoma typically appears as a painless, slowly growing, firm mass, most patient may be aware of the lesion for many months or years before seeking a diagnosis [3].

Presence of unusual large lesion in hard palate may often lead to diagnostic, surgical and reconstruction dilemma regarding its clinical and histopathological characteristics. Case management difficulty increased with immunocompromised condition such as Systemic Lupus Erythematosus. Surgical management of maxillary defects is one of the most difficult challenges related to complex anatomy and immense vascularity and difficult surgical access in narrow area causing high level of psychological and physical trauma, major discomfort in patients. High risk of wound dehiscence, bone necrosis, massive bleeding, stomatology problem and speech impairment related to velar musculature disarrangement [6,7]. Comprehensive approach in case management must be done to achieve a definite diagnosis and formulating the most suitable treatment plan.

This case series aim to present successful minimal invasive surgical and post-operative surgical defect approach of extensive pleomorphic adenoma in hard palate emphasizing on maxillary anatomical aspect, tumor pathology characteristic, basic wound healing mechanism, and comprehensive multidisciplinary management of comorbidity.

## 2 Case report

**Fig. 1:**
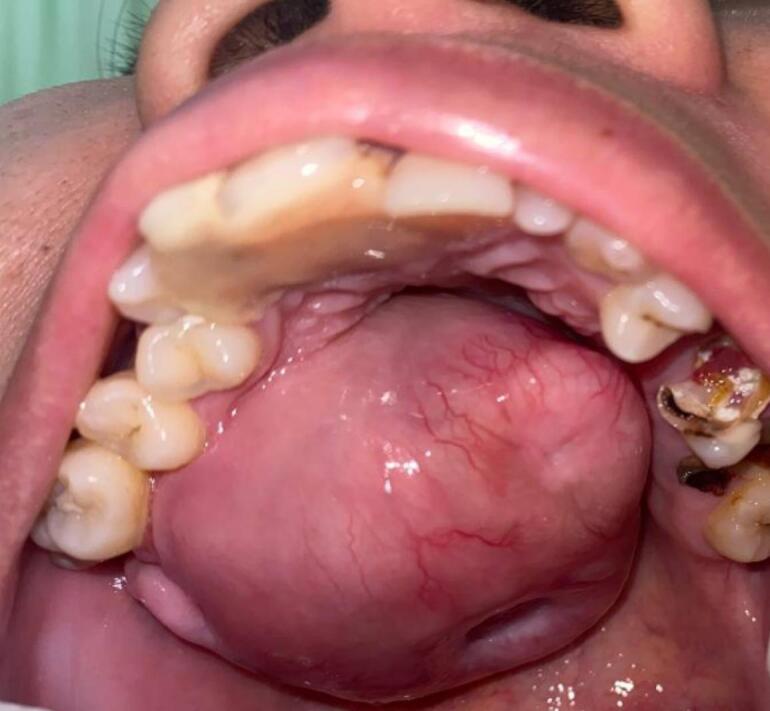
Firm mass on hard palate.

**Fig. 2:**
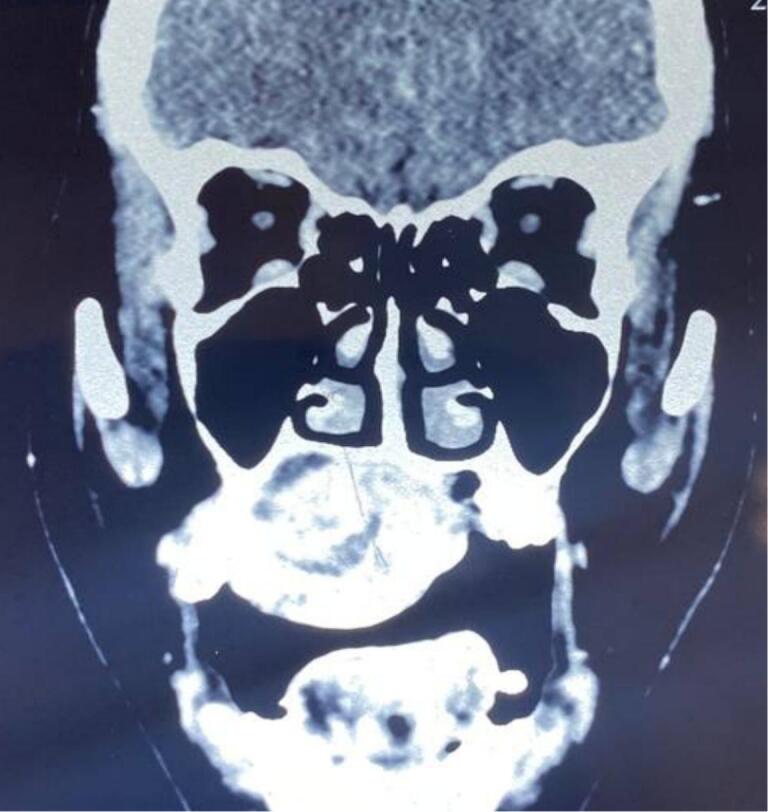
Coronal CT scan shoes expansive mass involving hard palate.

First case report was female 38 years old with chief complaint of slow growing mass in palate since 17 years ago. Starting as a small bump gradually increase in size causing difficulty in speaking, swallowing, chewing and eating, patient never seek medical treatment. Physical examination shows no extraoral facial asymmetry tenderness and enlargement of right submandibular and right and left mid jugular lymph node. Intraoral inspection found approximately 5 × 4 × 3 cm mass at right side of hard palate expanding beyond median palatine suture to left side and extending to posterior part to soft palate with diffuse border (Fig. 1). Endophytic area was observed in posterior part of lesion, mass was firm in consistency, fixed, smooth surface, nonpulsating and painless on palpation. Painless mobility of right maxillary first premolar until first molar and alveolar bone was found during palpation. Computed Tomography (CT) radiograph (Fig. 2) shows enhancing soft tissue mass with necrotic component without invasion into adjacent structures and no bony destruction. Fine Needle Aspiration Biopsy result was cell with oval, round, and spindle eccentric nucleus, with no atypic and dysplatic cell observed.

**Fig. 3:**
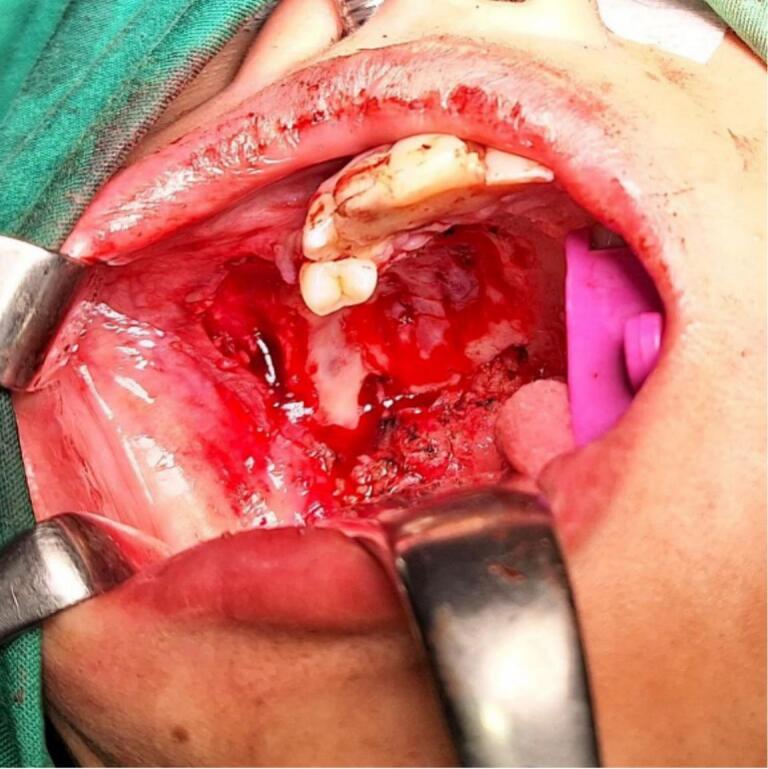
Immediate post-operative defect.

**Fig. 4:**
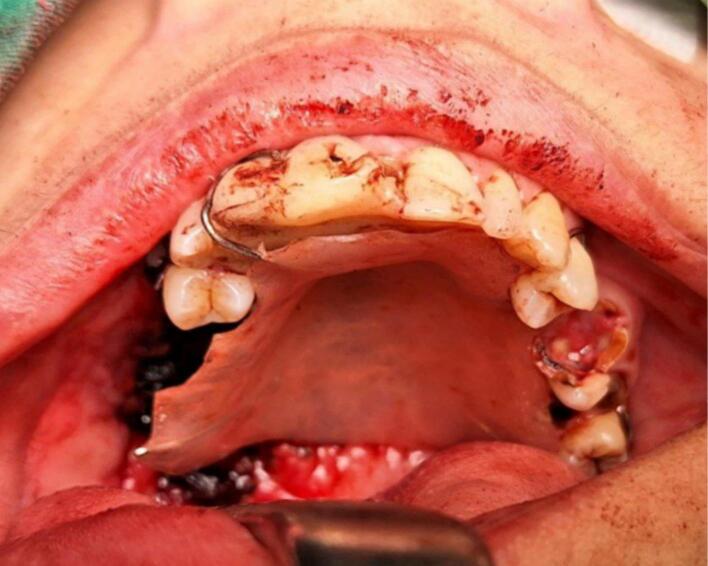
Surgical obturator placement in postoperative defect.

**Fig. 5:**
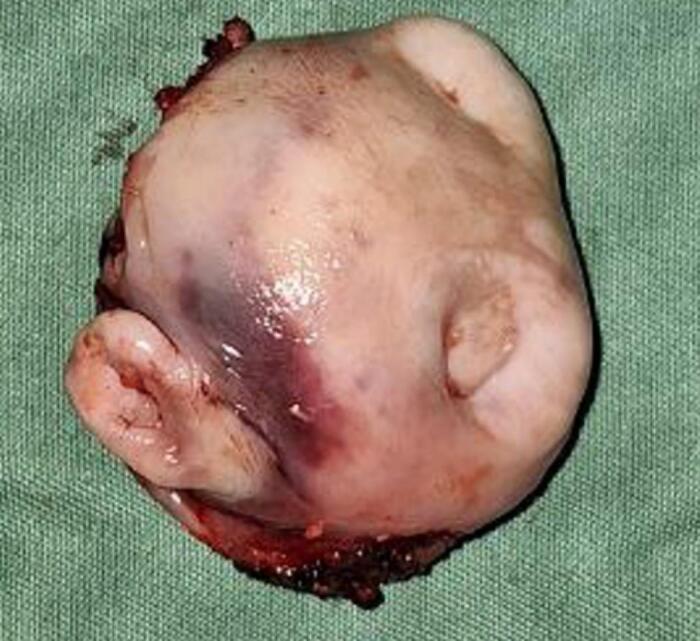
Gross specimen.

**Fig. 6:**
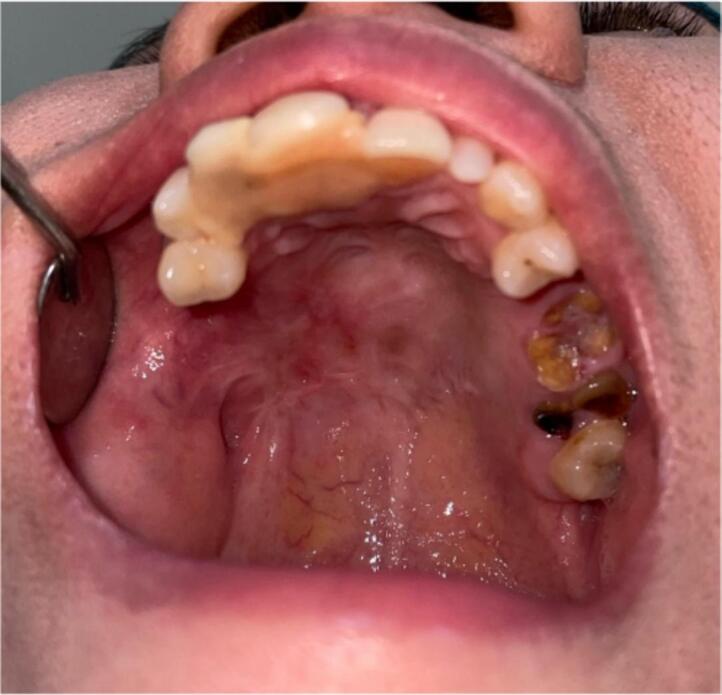
Post-operative defect third month.

**Fig. 7:**
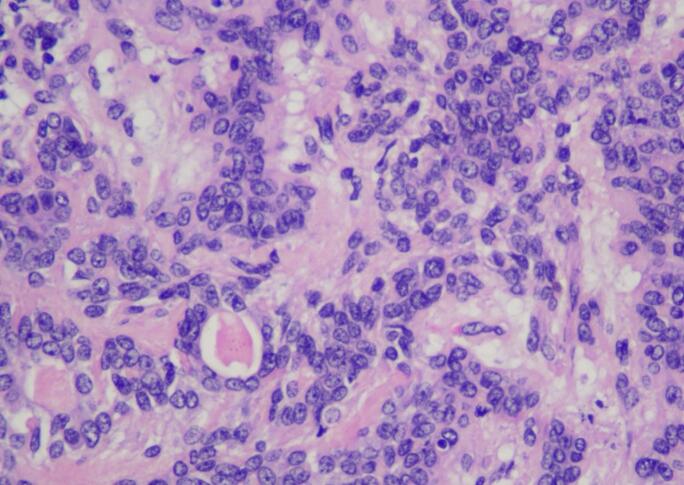
Post-operative histopathological examination.

Surgery was done with tumor excision, ostectomy, and surgical obturator placement under general anesthesia. During surgery, sessile wide base of tumor was identified and ligated to visualize precise area of incision. Peritumoral 1,5 mm marginal incision was made from right side area and extending the soft tissue incision to the left side. Bipolar cautery was used to control the bleeding in posterior part of right and left greater palatine artery and soft palate area near the tensor veli palatini, levator veli palatini muscle and palate aponeurosis. Massive hemorrhage occur during tumor excision and after mass removal, large denude area of palate was observed (Fig. 3). Infrastructure maxillectomy was done by removal of right maxillary bone from first premolar until posterior part of tuber maxilla using surgical saw and chisel. Palatal ostectomy was done using cutter cross bur with approximately 3-5 mm depth at the area involved by tumor and exposed area of nasal mucosa. Oxide regenerated cellulose hemostatic dressing applied into defect, immediate surgical obturator was placed to cover the surgical bed, gauze was filled between obturator and palate then secured with suture in the border of surgical obturator (Fig. 4). Gauze was removed 3 days after surgery. Post-operative Gross specimen of tumor measured 5 × 4 × 3 cm present solid mass without cystic fluid along with resected maxillary segment post-operative pathologic anatomy result was biphasic tumor growth was appeared on stroma with defined margin and without capsule consist of solid stranded epithelial component in accordance with pleomorphic adenoma (Fig. 5). Complete wound healing was present after 8 weeks (Fig. 6). Post-operative HPA result with 50× magnification showed ductal and myoepithelial cell in accordance with Pleomorphic Adenoma (Fig. 7).

**Fig. 8:**
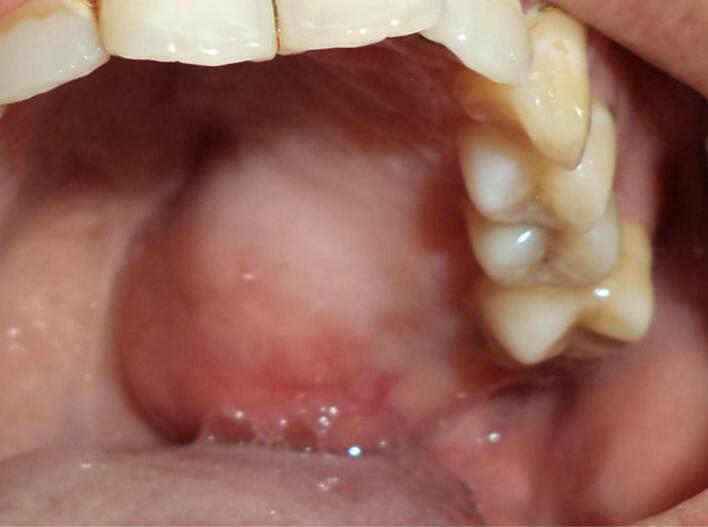
Firm mass on hard palate extending to soft palate.

**Fig. 9:**
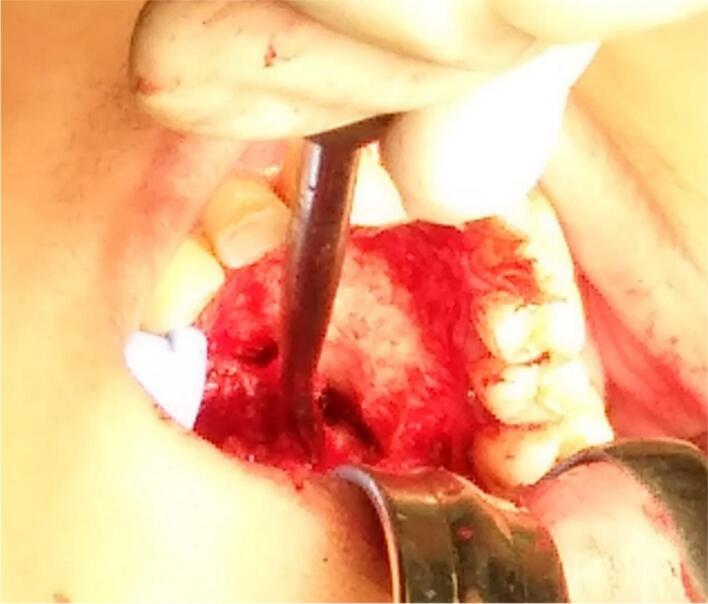
Immediate post-operative defect.

**Fig. 10:**
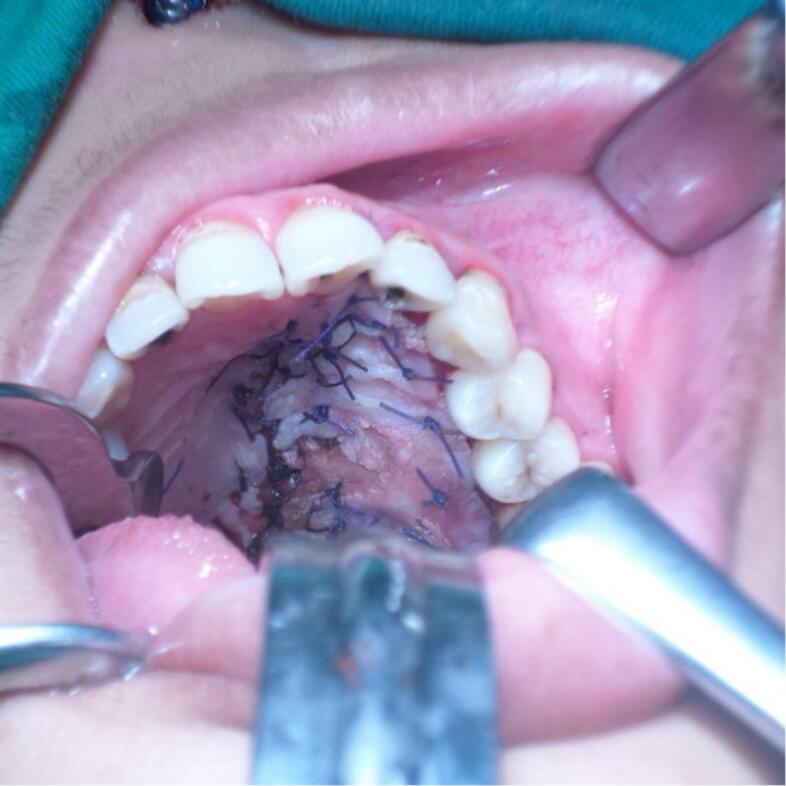
Immediate post-operative defect.

**Fig. 11:**
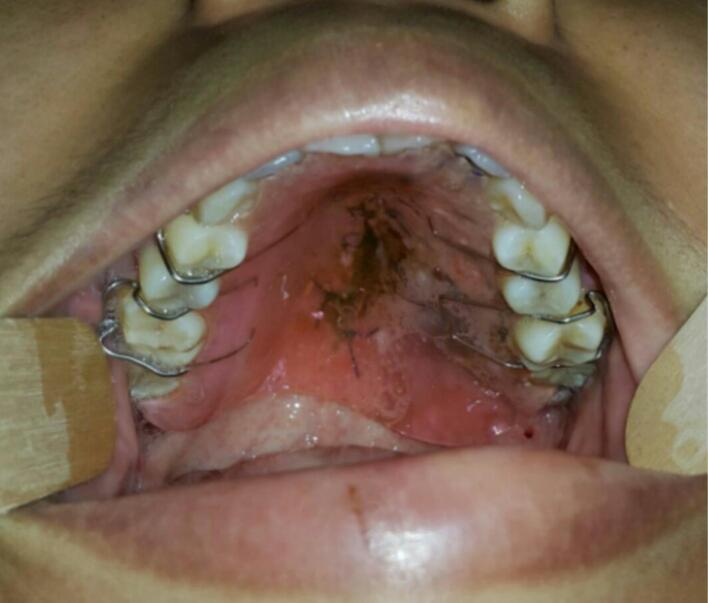
Surgical obturator placement in postoperative defect.

**Fig. 12:**
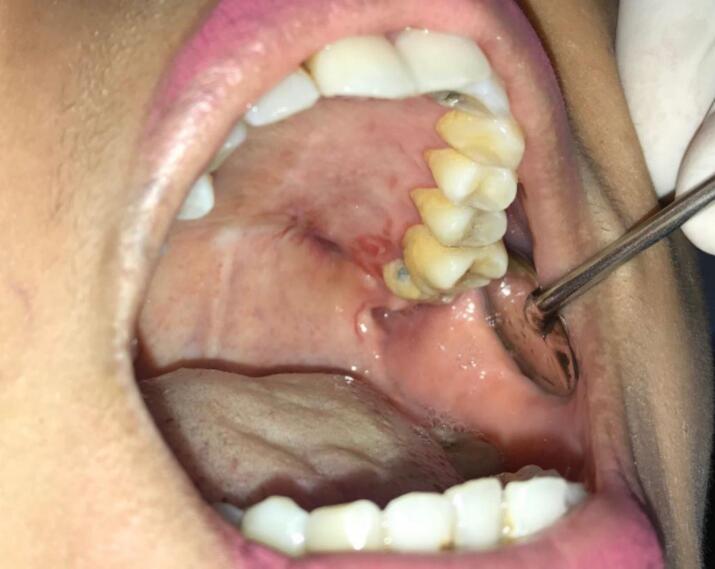
Post-operative defect third month.

Second case was 37 years old female with a chief complaint of swelling in the left side of hard palate since 5 years ago, it was small at first then gradually increased to it's present size causing speaking and swallowing difficulties. There is no recent significant weight loss. Past medical history was Systemic Lupus Erythematosus (SLE) since 3 years ago with routine consumption of Methylprednisolone. Intraoral inspection shows approximately 3 × 4 × 3 cm mass at left side of hard palate that not expanding beyond median palatine suture with diffuse border, mass was firm in consistency, fixed, smooth surface, nonpulsating and painless on palpation (Fig. 8). Computed Tomography (CT) radiograph shows enhancing soft tissue mass, attached to left side of hard palate with peritumoral vessel from maxillary artery without bone invasion. Serology Laboratory value related to SLE was observed and there are no sign of flare up. FNAB Result was benign salivary gland tumor with no malignant cells. Patient was admitted for tumor excision and with general anesthesia. Palatal mucoperiosteal flap was reflected to identify the mass, excision was done by completely separating encapsulated mass from palatal bone, ostectomy was done with 10 mm safe margin (Fig. 9), hemostatic dressing and gauze then applied into defect (Fig. 10). Primary closure was done and obturator was placed (Fig. 11). Gross specimen of excised solid mass was presented. Post-operative HPA 100× magnification showed duct and myoepithelial cell in eosinophilic background in accordance with pleomorphic adenoma. Long term follow up will be done for 5 years and complete epithelization of palatal wound was observed at third months after surgery (Fig. 12). Both of cases have been reported in line with the PROCESS criteria [26].

## 3 Discussion

The knowledge that pleomorphic adenoma can also appear in intraoral areas is important for the differential diagnosis of tumors in the head and neck region [8]. Several literature stated that intraoral pleomorphic adenoma is usually occurred as small lesion and considered rare to attain a size greater than 1–2 cm in diameter [6]. Contrary with finding in both of this cases that reach more than 3 cm in diameter causing diagnostic dilemma if it was benign or malignant. Regardless of the site of origin, Pleomorphic Adenoma typically appears as a painless, slowly growing, firm mass and most patient may be aware of the lesion for many months or years after reached considerably large size before seeking a diagnosis and this statement was in accordance with both of this cases which in the first case it was realized after 17 years and the second case was 5 years prior [3,9].

First crucial step in managing pleomorphic adenoma was to establish correct diagnosis, the most suitable treatment plan and rule out malignancy possibility. The diagnosis of Pleomorphic Adenoma is based on past medical history, clinical examination, with the main clinical sign was an increase in volume and size in hard palate extending to soft palate [10]. Both of cases presented here was painless slow growing mass more than 2 years ago, without significant weight and appetite loss, and no regional lymph node enlargement. Differential diagnosis for both cases was Ossifying Fibroma, Adenomatoid Odontogenic tumor, Mucoepidermoid Carcinoma, Polymorphous Adenocarcinoma. Physical and clinical examination found suspicious sign of malignancy in both of cases since extensive immobile, fixated mass and presence of ulceration was present in hard palate. In this case, ulceration in the lesion was attributed to chronic occlusal indentation of tooth during mastication. Pleomorphic adenomas of the palatal minor salivary glands remain a rather rare clinical condition, and it is unusual to infiltrate the adjacent palatal bone [4]. Both of this cases exhibit no bone invasion from CT scan but clinically in first case, there are right maxillary alveolar and tooth mobility with suspicion of bone invasion. Bone invasion is an important sign to determine aggressive and invasive behaviour of tumor [9]. If the lesion involves the bone then the periosteum and bone are to be included in the surgical procedure. Deep infiltration, bone involvement and perineural spread suggest malignancy that must be confirmed by several examination and diagnostic modalities [12]. Considering the complexity of the regional anatomy, diagnosis of bone invasion not only helps with the jaw resection decision or less conservative surgical approach, but also helps with discovering hidden or uncertain malignancies or aggressive lesion [13]. Most of literature stated that Pleomorphic adenoma cannot invade bone, this is not caused by bony invasion but rather by the inelasticity of the palatal mucosa, which becomes distended by the tumor mass and may eventuate in a cupped out resorption of bone by activating RANK, RANKL, OPG mechanism [11].

Several Radiodiagnostic and Histopathological examination are available to established diagnosis of Pleomorphic Adenoma. Combination of proper preoperative imaging and cytology or histopathology testing should be evaluated to formulate the most suitable treatment plan [4]. Tumor appearance could be detected by Computed Tomography (CT) or Magnetic resonance imaging (MRI). CT result of pleomorphic adenoma usually appears as smoothly marginated or lobulated homogeneous soft tissue density globular mass. Necrosis can be seen in larger masses. Few foci of calcification are common. This appearance was noted in both of this case [14]. Any misdiagnosis by the imaging techniques could induce a certain amount of maxillary bone loss and seriously influence the life qualities [13]. The definitive diagnosis will be determined by histopathological analysis [10]. Fine Needle Aspiration Biopsy (FNAB) is used as preoperative histopathologic diagnostic in both of this case to differentiate malignancy and benign lesions. These procedures are associated with very low tumor seeding rates [14]. FNAB can determine whether the tumor is malignant in nature with an approximate sensitivity of 90% [15]. Incisional biopsy was avoided in this case because pleomorphic adenoma has pseudopodic microscopic extension in surrounding normal tissue encapsulated in false capsule, incisional biopsy could trigger spillage of tumor cells into deeper tissue [2]. Before surgery was executed, pre-operative patient optimization and comorbidity must be regulated. Pre-operative treatment plan was more complex in second case because of history of Systemic Lupus Erythematosus (SLE) with routine consumption of methylprednisolone. It could present as major challenges to the anesthesiologist and surgeon because of risk of organ damage, septic shock, severe infection, deep vein thrombosis, pulmonary embolism and major acute coronary events [16]. This patient was consulted and co-treated with rheumatologist to establish optimal corticosteroid dose and duration and minimize the adverse effects on wound healing while still adequately treating the underlying condition. Methylprednisolone could impair all major phase of the wound healing. In this case corticosteroid therapy was still continued with normal dose 4 mg per day and must not to exceed 40 mg per day. Abrupt cessation of glucocorticoids or the stress response associated with surgery could precipitate Addisonian crisis because of hypothalamic pituitary axis (HPA) suppression [17].

Principle treatment plan for both cases was complete eradication of tumor to avoid reccurency and minimally invasive approach to preserve vital anatomical structure and quality of life. Surgical treatment for both cases was tumor excision with a surrounding cuff of normal mucosal tissue and involved bone. Most of intraoral pleomorphic adenoma usually does not recur after adequate surgical excision. Most recurrences can be attributable to inadequate surgical techniques that leaving behind microscopic pseudopod like extensions, or tumor spillage [18]. When dealing with large lesions of maxillary tumor, many authors report use of extraoral approaches. Contrary to that opinion, we decided to do intraoral approach because the lesion does not expand into surrounding bone. Extraoral approach such as the Weber Ferguson incision provides suitable surgical exposition, but it possesses the disadvantage of causing external scarring [9]. Complex anatomy of hard and soft palate make this anatomical region one of the hardest to reconstruct and rehabilitate, especially to restore the function of the soft palate. The main objective of reconstruction should be using the simplest technique to restore and maintain the separation between the oral and nasal cavities and thereby re-establishment of function [19]. Reconstructive surgery of the palatal defect may be achieved by using local, loco regional tissues such as free buccal mucosal graft (FBMG), buccal mucosal flap (BMF), or a buccinator flap (BMMF) with or without containing the facial artery. Larger defects of the soft palate can make a free flap reconstruction necessary. Using a radial forearm flap (RFFF) can be limited by consecutive speech, dysphagia and therefore can lead to relatively poor results [25].

Both cases here were treated with minimal invasive surgical approach with advantages such as minimum donor site morbidity, reduced length of surgery, being well tolerated by patients, simplicity to perform, repair with similar mucosal tissue, and avoidance of external scars with acceptable aesthetic results. For first case, mucoperiosteal local flap is insufficient to close the defect since it was exposed large area of raw surface in hard palate. Most of periosteum was excised with tumor, right side greater and minor palatine artery is not preserved and we have to rely on the remaining left greater palatine artery and antero-posterior branch of ascending palatine artery from posterior. Main consideration in this case was the defect greater than one third of the hard palate is challenging to reconstruct with local flaps [10,19]. A range of reconstructive techniques have been developed to overcome these difficulties. Exposed raw surface of hard palate was covered by antibiotic gauze packings, oxidized cellulose and or prefabricated acrylic stent or obturator to avoid hematoma and dead space formation, reduce postoperative discomfort and allowing healing by secondary intention [21].

For the second case, we used the whole of local mucoperiosteal flap based on one greater palatine artery to cover the area. Anatomically, it enter the mucoperiosteum at greater palatine foramen with greater palatine nerve to forms the major blood supply, so one pedicle can sustain most of the mucoperiosteum over the palate. The advantage of local mucoperiosteal flap is its vascularity, adequate availability and location. Rich maxillary vascular capillary network (Vascular macronets described by Meher) allows whole of the mucoperiosteum to be elevated based on one greater palatine artery [7]. The challenge was to recreate the mucosal lining without altering soft palate function. The result for both cases was good and acceptable. No wound dehiscence, intact oral mucosa was observed and no reccurency.

Key factor to achieve satisfactory result for these cases was understanding of wound healing mechanism and avoid dead space formation. A surgical stent or obturator is a device used to apply pressure on the soft tissues to facilitate healing and prevent cicatrization or collapse by providing support for the anastomosed structures. It can effectively prevent wound contamination, reduce infection susceptibility, and promote tissue healing [22]. In the knowledge that clot stability underpins successful wound healing, various dressing materials have been used in attempts to protect the palatal clot until healing is achieved [23]. Basic mechanism of palatal wound heals in four partially overlapping phases such as hemostasis, inflammatory, granulation, and maturation. In first case, secondary wound healing was intended because the large size of tumor. Palatal soft tissue healing by secondary intention is characterized by a larger tissue deficit that requires a longer healing time and is susceptible to a greater risk of infection or scarring. During the hemostatic phase, more pronounced inflammation is noted in wounds healing by secondary than by primary intention because more necrotic debris, exudate, and fibrin must be removed. Secondary intention wound healing predominantly involves granulation tissue formation; epithelialization only occurs when enough granulation tissue fills the injury site. After 5 days, an inflammatory infiltrate persists, but there is active migration of cells from the basal epithelia. Myofibroblast dependent wound contraction prevails in sites healing by secondary intention [24].

Based on wound healing mechanism, we used Mechanical protection methods for both cases, such as the custom-made acrylic obturator plate or stent are still considered as effective method to control palatal bleeding and aided secondary wound healing process. Iodoform embedded oxidized cellulose dressings and cross-over suturing technique, though hard to attain, are also used by several clinicians to keep the clot in place for longer duration.

Systematic review stated that both obturator dan flaps might be effective in rehabilitating the function of maxillary defect. Although many experts feel that obturators should only be applied in small defects. Several studies showed no significant difference was found in demonstrating that obturators might works as effective as flaps in several large defect in terms of healing promotion, oral hygiene, comfortability and superior in speech rehabilitation function [6]. It has been shown in both cases to be efficient in achieving a significant reduction in pain, prevent infection, discomfort, bleeding and permit socialising and routine daily work. This method will relief problems such as friction with palatal mucosa during function that could result in clot dislodgement causing bleeding associated with pain, impairment of healing and difficulty in speaking [23]. Removable obturator is used for 6 weeks because complete epithelialization is usually achieved in 3–5 weeks with maturation phase and tissue remodelling. Obturator permanently removed after wound healing epithelization assessment was done with hydrogen peroxide irrigation method by applying it to the palatal wound area. The absence of bubbling suggests that hydrogen peroxide has not been able to diffuse into the connective tissue and liberate oxygen, meaning that total epithelialization is present [24].

## 4 Conclusion

Comprehension of pleomorphic adenoma pathological characteristic, basic wound healing mechanism, anatomy, perioperative management are paramount to achieve successful tumor eradication with minimal invasive surgical approach and preserve patient's quality of life.

## Ethical approval

Ethical Approval is exempt/waived at our institution as long as patient's inform consent was gained and approved.

## Funding

N/A

## Author contribution

Nining Dwi Suti Ismawati: Case writing, supervision, visualization, investigation, drafting, editing, revising and reviewing;

Andreas Pratama Nugraha: Case writing, visualization, conceptualization, methodology, editing,

Ronny Baehaqi: conceptualization, methodology;

Irham Taufiqurrahman: conceptualization, methodology.

## Guarantor

Nining Dwi Suti Ismawati and Andreas Pratama Nugraha is the person in charge of the publication of our manuscript.

## Research registration number

1. Name of the registry: none
2. Unique identifying number or registration ID: none
3. Hyperlink to your specific registration (must be publicly accessible and will be checked):

## Consent

Written informed consent was obtained from the patient or/and guardian for publication of this case report and accompanying images. A copy of the written consent is available for review by the Editor-in-Chief of this journal on request.

## Conflict of interest statement

N/A
